# Quality Assessment of Systematic Reviews on Gluteal Augmentation Using a Measurement Tool to Assess Systematic Reviews (AMSTAR-2)

**DOI:** 10.1093/asjof/ojag162

**Published:** 2026-09-03

**Authors:** Elisa Bertulla, Edoardo Raposio

## Abstract

Gluteal augmentation has become increasingly popular in aesthetic surgery, with fat grafting, implants, and fillers as the main techniques. This study aims to assess the methodological rigor of available reviews using A Measurement Tool to Assess Systematic Reviews 2 (AMSTAR-2) and to explore associations with publication characteristics, including citations, journal impact factor, year of publication, and PRISMA adherence. A systematic literature search of PubMed, Scopus, and the Cochrane Library was performed. Two authors independently screened studies and extracted data. Methodological quality was assessed with AMSTAR-2. Correlations between AMSTAR scores and publication features were evaluated using Spearman's rank correlation, and differences by PRISMA adherence were tested with the Mann–Whitney *U* test. Interrater reliability was assessed with Cohen's kappa. Thirteen systematic reviews were included. Most focused on safety and complication rates of fat grafting vs implants. According to AMSTAR-2, 11 reviews were rated “critically low” and 2 “low.” Spearman correlations indicated a moderate positive association between AMSTAR score and year of publication (*r* = 0.46) and a weak-to-moderate association with the number of included studies (*r* = 0.39). No significant associations were observed with journal impact factor or citation count. PRISMA adherence was reported in 69% of reviews but did not significantly affect AMSTAR scores (*P* = .536). Although systematic reviews on gluteal augmentation are increasing, their methodological quality remains largely low. Future reviews should implement prospectively registered protocols, comprehensive search strategies, and rigorous risk-of-bias assessment to provide reliable guidance for clinical practice.

**Level of Evidence:** 3 (Therapeutic) 
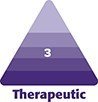

In recent years, gluteal augmentation has seen a marked increase in popularity within aesthetic plastic surgery, providing patients with increased volume, improved contour, and greater projection.^1^ The 2 main techniques for buttock enhancement are fat grafting (also known as Brazilian butt lift) and implants, each with its own advantages and limitations.^2^ Recently, filler injection has also gained popularity as a minimally invasive option for restoring gluteal volume.^3^

The morphological features associated with an aesthetically pleasing female buttock are multifactorial and extend beyond the overall curvature, the degree of “hourglass,” and the ideal waist-to-hip ratio of 0.7.^4-6^ For example, some analyses suggesting that proportions based on the golden ratio further contribute to perceived attractiveness.^7^ Additionally, the so-called “arrow effect”—created through targeted sculpting of the sacral triangle, upper gluteal quadrants, and dimples of Venus—plays an important role in overall back and gluteal aesthetics and can be further refined by considering the line of maximum lateral thigh projection.^8-12^

With the rising demand for buttock surgeries, it is crucial to evaluate which techniques provide the safest outcomes. High-quality systematic reviews and meta-analyses, assessed using tools such as A Measurement Tool to Assess Systematic Reviews 2 (AMSTAR-2), are essential for guiding evidence-based clinical decision making. AMSTAR has been applied in previous studies to assess the quality of systematic reviews and meta-analyses about breast augmentation and breast reconstruction.^13-15^ However, the quality of systematic reviews and meta-analyses specifically addressing gluteal augmentation has not yet been evaluated in the existing literature, to the best of our knowledge. The primary objective of this study was to evaluate the methodological quality of published reviews, whereas the secondary objective was to investigate the relationship between review quality and variables including citation count, journal impact factor, year of publication, and adherence to the PRISMA reporting guidelines.

## METHODS

This systematic review was conducted in accordance with the PRISMA reporting guideline.^16^ This study was prospectively designed, and its protocol was registered on Open Science Framework registries (https://osf.io/wkf4a).

### Search Strategy

A comprehensive literature search of PubMed, Scopus, and the Cochrane Library was performed in March 2026 to identify all systematic reviews and meta-analyses using key terms that pertained to gluteal augmentation. The search strategies for each database are available in the Appendix. Studies with duplicate titles were removed. Two authors (E.B. and E.R.) independently screened title and abstract to assess eligibility. When information provided in the title and abstract was insufficient to assess eligibility, the full text of the articles was retrieved and evaluated. Full-text screening for inclusion was then independently performed by the authors. Any disagreements arising during the 2-stage screening process were resolved by discussion and consensus.

### Eligibility Criteria

Studies were eligible for inclusion if they focused on aesthetic gluteal augmentation and were classified as systematic reviews or meta-analyses in the title or manuscript text. Reviews that did not explicitly identify themselves as systematic but reported the use of a systematic literature search to identify relevant studies were also considered eligible for inclusion. Studies were excluded if they were not published in English, involved nonhuman research, or represented other publication types, including case reports, narrative reviews, expert opinions, editorials, study protocols, or conference abstracts.

### Data Collection and Analysis

Data extraction was performed independently by 2 reviewers (E.R. and E.B.), with any disagreements resolved through discussion until consensus was reached. The methodological quality of the included reviews was evaluated using the AMSTAR-2 instrument. In addition, data were collected on journal name and impact factor (Web of Science, Clarivate Analytics, Philadelphia, PA), publication year, corresponding author's country of affiliation, adherence to the PRISMA reporting guidelines, citation count (Web of Science), review topic, and the number of studies included. The principal findings and conclusions reported by each review were also extracted and synthesized.

### Quality Assessment

The methodological quality of the included reviews was evaluated using the AMSTAR-2 instrument.^13^ This validated appraisal tool is specifically intended to assess the methodological rigor and reporting transparency of systematic reviews of healthcare interventions. AMSTAR-2 comprises 16 assessment domains, of which 7 are designated as critical for establishing the overall level of confidence in a review's findings. Each domain is scored as “Yes,” “No,” or “Not Applicable,” depending on whether the corresponding methodological criterion has been fulfilled (Table 1). Two reviewers independently assessed each criterion and recorded their judgments as “Yes,” “No,” or “Not applicable.” Any disagreements were subsequently resolved by discussion and consensus.

**Table 1.** AMSTAR-2 Criteria

AMSTAR criteria	Description	Critical item
1	Clear research question and inclusion criteria (PICO)	No
2	Presence of a previous study design	Yes
3	Explanation of the inclusion methods of the studies	No
4	Comprehensive literature search strategy	Yes
5	Duplicate study selection	No
6	Duplicate data extraction	Yes
7	List of studies excluded provide	Yes
8	Adequate detailed description of the included studies	No
9	Risk-of-bias assessment	Yes
10	Source of findings for the studies reported	No
11	Appropriate methods for statistical combination of results	Yes
12	Consideration of risk of bias on results	No
13	Consideration of risk of bias in interpretation of the results	Yes
14	Explanation of heterogeneity	No
15	Risk of publication bias assessment	Yes
16	Conflict of interest stated	No

AMSTAR-2, A Measurement Tool to Assess Systematic Reviews 2.

The overall level of confidence assigned to each review was categorized as high, moderate, low, or critically low, according to the number and severity of methodological weaknesses identified. This rating is determined by the presence of both critical and noncritical flaws, with critical weaknesses representing methodological deficiencies that may substantially compromise the reliability of a review's conclusions. The critical domains evaluated by AMSTAR-2 include protocol registration before review initiation, the comprehensiveness of the literature search, justification for excluded studies, assessment of the risk of bias in individual studies, use of appropriate methods for meta-analysis, consideration of the risk of bias when interpreting the findings, and evaluation of potential publication bias.

Microsoft Excel (Microsoft, Redmond, WA) was used for data management, tables and figures construction, and statistical analysis. Pairwise correlations between AMSTAR score and citation number, impact factor, publication year, and number of studies included were evaluated using the Spearman correlation coefficient (*r*). Differences in AMSTAR score according to adherence to PRISMA guidelines were analyzed using the Mann–Whitney *U* test. *P* values <.05 were considered statistically significant. Interrater reliability between the 2 reviewers was assessed using the Cohen's kappa statistic.

## RESULTS

### Search Results

The literature search retrieved a total of 39 records, of which 22 were identified as duplicates and removed (Figure 1). The remaining records underwent title and abstract screening, resulting in 16 studies being selected for full-text evaluation. Following assessment of the full-text articles, 3 additional studies were excluded, and the remaining studies were included in the final analysis. Ultimately, 13 studies were included in the review.^1-4,17-25^

**Figure 1.**
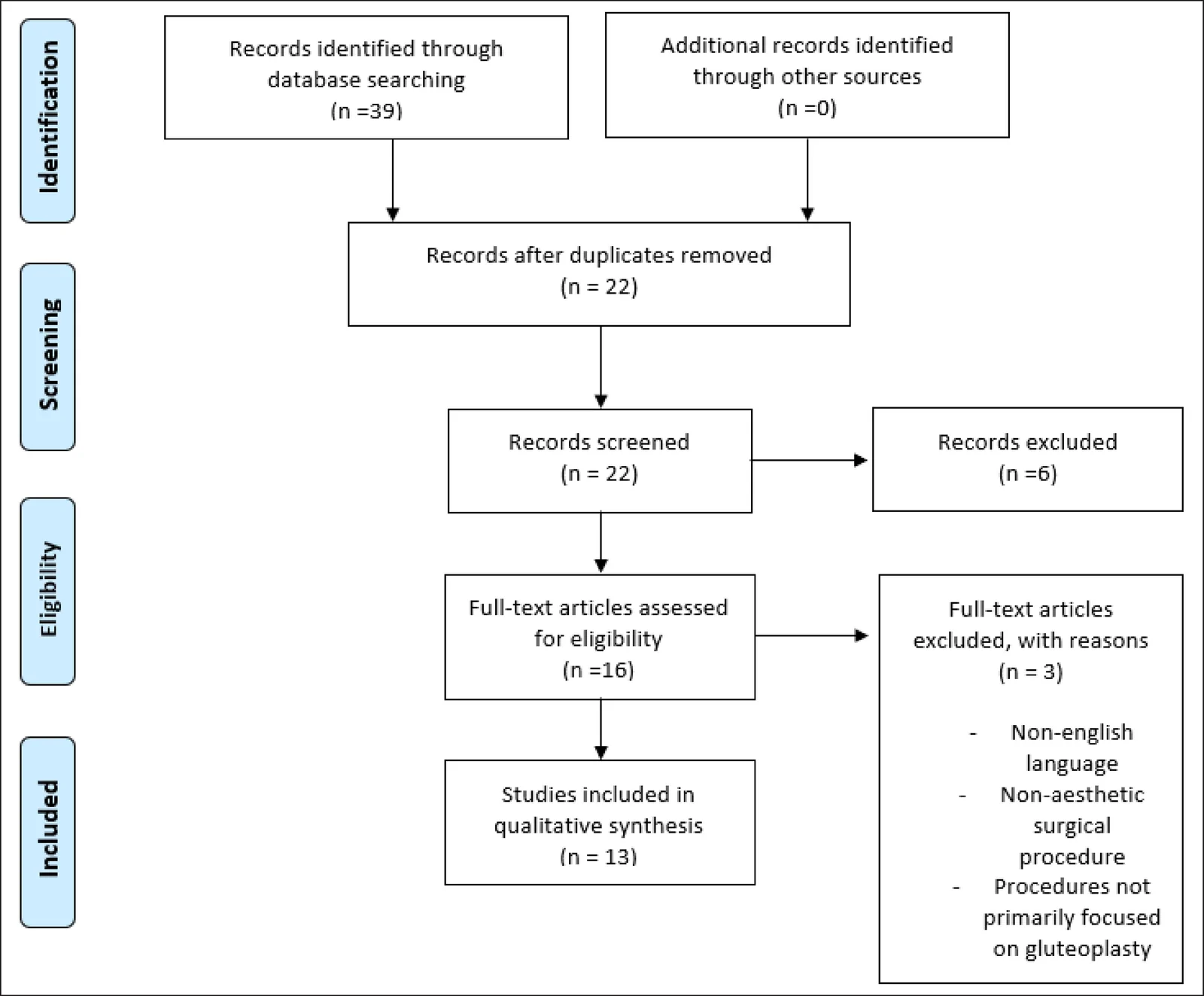
PRISMA flow diagram of study selection.

### General Study Characteristics

General study characteristics are summarized in Table 2. A total of 13 systematic reviews were included. Of these, 6 studies were affiliated with institutions in the United States, 3 in Poland, 1 in the United Kingdom, 1 in El Salvador, 1 in France, and 1 in Saudi Arabia. The main journals publishing these reviews were *Plastic and Reconstructive Surgery* (5 articles, Impact Factor 2.39), *Aesthetic Surgery Journal* (4 articles, IF 3.9), *Aesthetic Plastic Surgery* (2 articles, IF 2.32), *Annals of Plastic Surgery* (1 article, IF 1.5), and *JPRAS* (1 article, IF 1.58).

**Table 2.** Characteristics of Included Studies

Author, year	Journal	Impact factor	Country affiliation (corresp. author)	Total citation	No. of studies included	No. of patients included	PRISMA adherence	AMSTAR evaluation
Asserson et al (2019)^4^	*Annals of Plastic Surgery*	1.5	USA	25	134	4362	No	Critically low
Oregi et al (2023)^17^	*JPRAS*	1.58	UK	7	15	NR	Yes	Critically low
Safran et al (2020)^18^	*Aesthetic Surgery Journal*	3.9	USA	7	13	NR	Yes	Critically low
Elsaftawy et al (2025)^19^	*PRS*	2.39	Poland	47	32	2682	Yes	Low
Sinno et al (2016)^20^	*PRS*	2.39	USA	104	44	2375	No	Critically low
Condè-Green et al (2016)^21^	*PRS*	2.39	USA	71	19	NR	No	Critically low
Elsaftawy et al (2025)^19^	*PRS*	2.39	Poland	NR	38	22,151	Yes	Low
Dorfman et al (2022)^22^	*PRS*	2.39	USA	4	8	NR	Yes	Critically low
Flores-Lima et al (2013)^23^	*Aesthetic Plastic Surgery*	2.32	San Salvador	27	30	NR	No	Critically low
Mortada et al (2023)^3^	*Aesthetic Plastic Surgery*	2.32	Saudi Arabia	10	10	168	Yes	Critically low
Milani Reis et al (2025)^24^	*Aesthetic Surgery Journal*	3.9	USA	NR	4	6235	Yes	Critically low
Elsaftawy et al (2025)^19^	*Aesthetic Surgery Journal*	3.9	Poland	27	13	NR	Yes	Critically low
Carloni et al (2016)^25^	*Aesthetic Surgery Journal*	3.9	France	16	28	1380	Yes	Critically low

JPRAS, *Journal of Plastic, Reconstructive and Aesthetic Surgery*; PRS, *Plastic and Reconstructive Surgery.*

The publication years ranged from 2016 to 2026, with a relatively even distribution of articles across the years. Citations per article varied from 4 to 104, with an average of 31.3 (in 2 cases, citation data were not available in Web of Science). The number of studies included within each review ranged from 4 to 134. The total number of patients ranging from 168 to 22,000. In 6 reviews, the total number of patients included across studies was not reported.

The findings of included studies were synthesized based in Table 3. Most reviews focused on safety and complication rates of gluteal augmentation procedures, comparing prosthetic implants with autologous fat grafting. One review focused on gluteal augmentation with hyaluronic acid, one on the role of corticosteroids in preventing fat embolism, one proposed a new buttock classification system, and one addressed ultrasound-guided fat grafting.

**Table 3.** Summary of Conclusions of Each Review Article Included in the Study

Author, year	Objective of the study	Summary of conclusions
Asserson et al (2019)^4^	Complication rates between implant, fat grafting, and local flap	Implants had the highest rate of complications (31.4%), followed by flaps (23.15), whereas fat grafting showed the lowest rate (6.8%).
Oregi et al (2023)^17^	Safety of implant and fat grafting	Fat grafting leads to fewer complications and allows greater contouring.
Safran et al (2020)^18^	Role of corticosteroid prophylaxis for the prevention of microscopic fat embolism syndrome	A single perioperative EV dose of methylprednisolone may be appropriate although the safety. The implication of this therapy on wound healing and fat graft survival need to be discuss.
Elsaftawy et al (2025)^19^	Complication following primary implant-based gluteal augmentation	Textured implants had higher complication rates. Intramuscular and submuscular placements showed lower complication rates.
Sinno et al (2016)^20^	Safety and efficacy of gluteal augmentation	The overall complication rate with autologous fat grafting (9.9%) is lower than that with silicone buttock implants (21.6%).
Condè-Green et al (2016)^21^	Fat grafting for gluteal augmentation	Fat grafting is an effective and predictable way to remodel the gluteal region; however, the procedure is not without risks. Avoiding gluteal vessel damage may prevent most feared complications, such as fat embolism.
Elsaftawy et al (2025)^19^	Fat grafting for gluteal augmentation	Fat grafting is generally safe. Subcutaneous-only injections and ultrasound guidance are associated with lower rates of complications. The use of lipoaspirate additives and drains did not consistently lower complication rates.
Dorfman et al (2022)^22^	Proposed a novel classification system for buttock	This review identifies a gap in the literature regarding gluteal size and shape classification. The authors propose a novel system that is characterized by 2 main factors: projection and its relation to the ASIS and infragluteal fold. The 3 category types are defined according to the position of the point of maximal gluteal projection. Within each type, there are 3 subcategories—low, moderate, and plus—that relate to volume projection.
Flores-Lima et al (2013)^23^	Surgical pocket location for implants	The pocket locations for gluteal augmentation surgery with implants have the lowest complication rates are the following: intramuscular XYZ method (13.18%), submuscular (17.60%), intramuscular (18.05%), and subfascial (54.84%).
Mortada et al (2023)^3^	Hyaluronic acid injection for gluteal augmentation	Mean volume of HA injected for optimal gluteal augmentation was 206.71 mL. Of the studies included, 2 reported major complications, 5 patients experienced bruising and erythema, 5 patients experienced effusion and inflammation at the injection site, 3 patients experienced gel dislocation, and 1 patient experienced irregular contours.
Milani Reis et al (2025)^24^	Ultrasound-guided gluteal fat grafting	The findings indicate that ultrasound-guided gluteal fat grafting is associated with low complication rates and no reported serious adverse events, such as death or fat embolism, reinforcing its role as a safer technique for buttock augmentation.
Elsaftawy et al (2025)^19^	Combined implant and lipoplasty techniques in primary gluteal augmentation	The pooled implant removal was higher in the submuscular plane (1.69%) than the intramuscular plane (0.51%). The intramuscular plane had higher dehiscence and hematoma rates, whereas submuscular placement carried a greater risk of sciatic neuropathy (3.91%). Drain use was associated with higher seroma rates but lower infection and hematoma incidence.
Carloni et al (2016)^25^	Factors predictive of postoperative complications in circumferential contouring of the lower trunk	Lower body lift-related techniques were associated with a higher rate of overall complications than belt lipectomy-related techniques. No difference in complication rate was shown when performing a gluteal augmentation with flap. Because of insufficient data reported in the studies, risk factors for postoperative complications could not be assessed.

ASIS, anterior superior iliac spine; EV, endovenous; HA, hyaluronic acid.

The conclusions across studies were consistent: fat grafting was associated with lower complication rates compared with prosthetic implants and generally provided satisfactory aesthetic results, although in some cases, implants were still necessary. Reviews agreed that the use of ultrasound guidance and exclusively subcutaneous injections reduced the complication rate. Regarding prosthetic implants, textured implants were associated with higher complication rates. The lowest complication rates were observed with intramuscular implantation, especially using the XYZ technique, and submuscular placement, whereas subfascial placement was the riskiest, showing the highest implant removal rates.

Adherence to guidelines and PRISMA was reported in 69% of the reviews (9 out of 13). According to AMSTAR-2 assessment, 11 reviews were rated as critically low and 2 as low. To assess correlations between AMSTAR scores and citation number, journal impact factor, publication year, and number of included studies, the ordinal AMSTAR ratings were converted into numeric values (high = 4, moderate = 3, low = 2, critically low = 1). Spearman's rank correlation (*r*) was used because of the ordinal nature of AMSTAR scores and the small sample size. The Spearman correlation coefficients between AMSTAR scores and the other variables indicate that the only moderate positive association was observed with the year of publication (*r* = 0.46). A weak-to-moderate positive correlation was observed with the number of included studies (*r* = 0.39), whereas no correlation was found with the journal impact factor (*r* = 0). Finally, the correlation with the number of citations was weakly positive (*r* = 0.30).

The Mann–Whitney *U* test was used to compare AMSTAR scores between studies adhering to PRISMA guidelines and those that did not. Studies following PRISMA had a median AMSTAR score slightly higher than those not following PRISMA; however, the difference was not statistically significant (*U* = 14, *z* = −0.62, *P* = .536). Cohen's kappa was 0.892, indicating almost perfect agreement between the 2 reviewers and high interrater reliability.

## DISCUSSION

This overview systematically appraised the current body of systematic reviews and meta-analyses addressing aesthetic gluteal augmentation, with a particular focus on their methodological quality as assessed by the AMSTAR-2 instrument. Overall, the methodological standard of the available secondary evidence was poor, with the vast majority of reviews demonstrating substantial methodological limitations. Specifically, 11 of the 13 included reviews were classified as critically low according to the AMSTAR-2 rating system, and the remaining 2 as “low.” None achieved a “moderate” or “high” level of confidence.

These findings highlight a substantial gap between the increasing popularity of gluteal augmentation procedures and the robustness of the evidence base synthesizing their outcomes. Over the past decade, as the demand for gluteal enhancement has grown globally, the number of published systematic reviews has also increased.^26^ However, this quantitative growth has not been accompanied by a parallel improvement in methodological quality.

### Methodological Quality and Clinical Implications

None of the included reviews achieved a moderate or high overall confidence rating based on the AMSTAR-2 criteria. All reviews exhibited at least one critical methodological weakness, a finding that, according to the AMSTAR-2 framework, precludes assignment of either a moderate or high level of confidence.

Notably, Item 7 (reporting the list of excluded studies) was never satisfied in any of the included reviews, which contributed substantially to the low overall confidence ratings. Other critical domains frequently unmet were Item 2 (previous protocol registration), absent in 8 of 13 reviews; Item 9 (risk-of-bias assessment), missing in 6 of 13; and Item 15 (publication bias assessment), not fulfilled in 8 of 13 reviews. Items 11 and 13 applied only to reviews that conducted a meta-analysis (7 of 13) and were not satisfied in 2 of these 7 reviews.

Importantly, despite the general agreement among the included reviews, this synthesis should not be interpreted as evidence that all conclusions are robust or directly transferable to clinical practice. Some conclusions may be influenced by the methodological limitations of the primary studies or of the included reviews. Therefore, clinical recommendations should be interpreted with caution.

### Temporal Trends and Factors Associated With Quality

The observed moderate association between publication year and AMSTAR score (*r* = 0.46), suggesting a slight improvement in methodological quality in more recent publications. This trend may reflect growing awareness of the value of evidence-based standards and clear reporting.

Similarly, the weak-to-moderate positive correlation observed between the number of included studies and AMSTAR score (*r* = 0.39) may indicate that broader reviews tend to demonstrate slightly stronger methodology, although the association remains limited. Notably, no correlation was found between journal impact factor and methodological quality (*r* = 0), indicating that publication in higher-impact journals does not necessarily guarantee greater methodological rigor. The weak positive correlation between citation count and AMSTAR score (*r* = 0.30) suggests that more frequently cited reviews tend to have slightly higher quality, although this relationship is modest. These results are in line with previous similar studies on breast reconstruction and breast augmentation procedures.^14,15^

Sixty-nine percent of reviews reported adherence to PRISMA guideline. The included studies span a broad publication period (2016-2026), covering both pre- and postimplementation phases of the PRISMA 2009 and its updated version, PRISMA 2020. Lack of reported adherence to PRISMA was observed across different time points (2 studies from 2016, 1 from 2019, and 1 from 2021), suggesting that variability in reporting practices may not be fully explained by publication year or guideline evolution but may indicate persistent inconsistencies in reporting across the literature.

Although 69% of reviews reported adherence to PRISMA guidelines, no statistically significant difference in AMSTAR scores was found between PRISMA-adherent and nonadherent studies (*P* = .536). This finding underscores the important distinction between reporting quality and methodological quality: compliance with reporting standards does not guarantee rigorous design or conduct. Additionally, these findings contrast with the results of similar studies by Yuan et al, Fleming et al, and Bole et al who noted that AMSTAR and PRISMA scores are significantly correlated.^15,27,28^ The discrepancy may be attributable to the limited number of included reviews. In our case, this finding underscores the distinction between reporting quality and methodological quality: formal adherence to reporting guidelines does not necessarily translate into rigorous study design and execution.

### Limitations and Future Directions

This overview has several limitations. First, inclusion was restricted to English-language publications, potentially introducing language bias and potentially excluding relevant evidence published in other languages. Second, the total number of eligible systematic reviews was relatively small (*n* = 13), limiting the statistical power of correlation analyses and reducing the ability to detect more subtle associations between methodological quality and publication characteristics. Additionally, the conversion of ordinal AMSTAR categories into numeric values, although necessary for statistical testing, represents a methodological simplification that may not fully capture the complexity of differences in methodological quality between reviews. A further limitation is that the assessment of reported adherence to the PRISMA guidelines did not account for the publication year of the included reviews or for the evolution from PRISMA 2009 to PRISMA 2020. Therefore, our findings should be interpreted as reflecting reporting practices across different publication periods, rather than uniform compliance with a single reporting standard.

The findings of this overview emphasize the need for future systematic reviews with prospectively registered protocols, comprehensive and reproducible search strategies, rigorous risk-of-bias assessment, and appropriate evaluation of publication bias.

## CONCLUSIONS

Although the number of systematic reviews on gluteal augmentation is expanding and demonstrates relative consistency in reported conclusions, its overall methodological quality remains predominantly low. Consequently, clinical decision making should therefore rely not only on published syntheses but also on critical appraisal of the strength and validity of the evidence underlying evidence. Future reviews should prioritize prospectively registered protocols and risk-of-bias assessment, including evaluation of publication bias, to ensure that conclusions can reliably guide safe and effective clinical practice.

## Supplementary Material

ojag162_Supplementary_Data
