# Supplementary material for: Quality Assessment of Systematic Reviews on Gluteal Augmentation Using a Measurement Tool to Assess Systematic Reviews (AMSTAR-2)
Source: Aesthet Surg J Open Forum. 2026 Sep 3;8:ojag162. doi: 10.1093/asjof/ojag162 (PMC13660799; doi:10.1093/asjof/ojag162)
Supplement: Supplementary file 1 [file ojag162_supplementary_data.docx]

**Appendix**

**Pubmed**

[Title/Abstract] ("gluteal augmentation" OR "buttock augmentation" OR "Brazilian butt lift” OR gluteoplasty OR "augmentation gluteoplasty" OR "gluteal contouring" OR "buttock contouring"

AND (systematic review [Publication Type] OR systematic review [Title/Abstract] OR meta-analysis [Publication Type] OR meta-analysis [Title/Abstract])

**Scopus**

("gluteal augmentation" OR "buttock augmentation" OR "Brazilian butt lift" OR gluteoplasty OR "augmentation gluteoplasty" OR "gluteal contouring" OR "buttock contouring”) AND ("systematic review" OR "meta-analysis")

**Cochrane**

("gluteal augmentation" OR "buttock augmentation" OR "Brazilian butt lift" OR gluteoplasty OR "augmentation gluteoplasty" OR "gluteal contouring" OR "buttock contouring") AND ("systematic review" OR "meta-analysis")
